# Performance optimization of energy-efficient solar absorbers for thermal energy harvesting in modern industrial environments using a solar deep learning model

**DOI:** 10.1016/j.heliyon.2024.e26371

**Published:** 2024-02-15

**Authors:** Ammar Armghan, Jaganathan Logeshwaran, S. Raja, Khaled Aliqab, Meshari Alsharari, Shobhit K. Patel

**Affiliations:** a Department of Electrical Engineering, College of Engineering, Jouf University, Sakaka, 72388, Saudi Arabia; b Department of Electronics and Communication Engineering, Sri Eshwar College of Engineering, Coimbatore, 641202, India; c Research and Development, Mr.R Business Corporation, Karur, 639004, Tamil Nadu, India; d Department of Computer Engineering, Marwadi University, Rajkot, Gujarat, 360003, India

**Keywords:** Thermal, Energy harvesting, Deep learning, Renewable energy, Solar absorber, Solar radiation

## Abstract

Thermal energy harvesting has seen a rise in popularity in recent years due to its potential to generate renewable energy from the sun. One of the key components of this process is the solar absorber, which is responsible for converting solar radiation into thermal energy. In this paper, a smart performance optimization of energy efficient solar absorber for thermal energy harvesting is proposed for modern industrial environments using solar deep learning model. In this model, data is collected from multiple sensors over time that measure various environmental factors such as temperature, humidity, wind speed, atmospheric pressure, and solar radiation. This data is then used to train a machine learning algorithm to make predictions on how much thermal energy can be harvested from a particular panel or system. In a computational range, the proposed solar deep learning model (SDLM) reached 83.22 % of testing and 91.72 % of training results of false positive absorption rate, 69.88 % of testing and 81.48 % of training results of false absorption discovery rate, 81.40 % of testing and 72.08 % of training results of false absorption omission rate, 75.04 % of testing and 73.19 % of training results of absorbance prevalence threshold, and 90.81 % of testing and 78.09 % of training results of critical success index. The model also incorporates components such as insulation and orientation to further improve its accuracy in predicting the amount of thermal energy that can be harvested. Solar absorbers are used in industrial environments to absorb the sun’s radiation and turn it into thermal energy. This thermal energy can then be used to power things such as heating and cooling systems, air compressors, and even some types of manufacturing operations. By using a solar deep learning model, businesses can accurately predict how much thermal energy can be harvested from a particular solar absorber before making an investment in a system.

## 1 Introduction

Thermal energy harvesting is a method of capturing and storing energy from the environment in order to be used as a power source. This type of energy harvesting has the potential to revolutionize the way we power our world [1]. Thermal energy harvesting can be used to provide energy for a variety of applications, from powering homes and businesses to powering cars, airplanes, and even spacecraft. Thermal energy harvesting works by capturing the heat generated by the environment and using it to generate electricity [2]. Heat is generated from sources such as the sun, industrial activities, and burning of fossil fuels. The heat can be captured in various ways, such as using a heat exchanger, or using a thermoelectric generator. The captured heat is then converted into electricity by a generator, and is then sent to a storage device such as a battery. The benefits of thermal energy harvesting are numerous [3]. It is a clean, renewable energy source that does not emit harmful pollutants into the atmosphere. It is also more efficient than traditional power sources, as it requires less energy to produce electricity. Additionally, it is a relatively inexpensive energy source that can potentially be used in a variety of applications [4]. Thermal energy harvesting also has the potential to revolutionize the way we power our homes and businesses. By using thermal energy harvesting systems, we can reduce our dependence on traditional energy sources such as coal and oil. Additionally, it can be used to provide energy in remote areas where traditional energy sources are not available [5]. Thermal energy harvesting has the potential to be a game-changer in the world of energy production and storage. While there is still a lot of research and development to be done, this type of energy harvesting has the potential to revolutionize the way we power our world. One potential application of the solar absorber is in the production of electricity [6]. By using the thermal energy that is generated by the absorber, it is possible to generate electricity using a turbine or other heat engine. This electricity can then be used to power homes and businesses, providing a renewable and cost-effective alternative to traditional electricity sources [7]. Another potential application of the solar absorber is in the production of hot water. By using the thermal energy generated by the absorber, it is possible to heat water to the desired temperature. This hot water can then be used for various applications, such as providing hot water for showers or heating a swimming pool [8]. The solar absorber is an essential component of thermal energy harvesting. It is responsible for absorbing solar radiation and converting it into thermal energy, which can then be used to generate electricity or provide hot water. As the demand for renewable energy sources increases, the solar absorber is likely to play an increasingly important role in the production of renewable energy [9].

A solar thermoelectric generator (STG) is a device that takes advantage of the heat of the sun to generate electricity. It works by harnessing the energy of the sun and converting it into electrical energy through a thermoelectric process [10]. This process involves the use of a thermoelectric material, which is heated by the sun and then used to generate electricity. The solar thermoelectric generator has a number of advantages over traditional methods of generating electricity. The STG is a renewable source of energy, meaning that it can be used without causing any harm to the environment [11]. Furthermore, it does not require any additional fuel or energy sources and is able to generate electricity in areas where other sources of electricity may be inaccessible. Another major benefit of the it’s cost effectiveness. It requires minimal maintenance and installation costs and can be used for a long time without any additional costs [12]. It does not generate any hazardous waste, making it a safe and efficient way to generate electricity. One of the drawbacks of it is not as efficient as other methods of generating electricity. It is limited in its capacity to generate electricity and is therefore used mainly in applications where other sources of electricity may not be available [13]. Despite its drawbacks, the solar thermoelectric generator has a number of advantages. It is a renewable source of energy, cost effective, and safe. It is also a versatile and efficient way to generate electricity in areas where other sources of electricity may not be available. For these reasons, it is a great option for those looking for a way to generate electricity without causing harm to the environment [14]. Energy harvesting is one of the most important challenges of modern times. With the increasing demand for energy, the world is looking for alternative sources of energy that are sustainable and renewable. One such source is STG. It is a device that converts solar radiation into electrical energy [15]. The main challenge in obtaining maximum energy harvesting using it is the efficiency of the device. Solar radiation is the primary source of energy for it, but the energy conversion rate of the device is quite low. Therefore, it is necessary to increase the efficiency of the device for maximum energy harvesting [16]. Another challenge is the cost of the device. It is expensive and its components are not easily available in the market. Moreover, the installation cost of the device is also high due to the complexity of the system. Therefore, the cost of the device should be reduced to make it more affordable for everyone. The third challenge is the storage of the energy generated by the device [17]. It stores the energy in the form of electrical energy, which is not suitable for long-term storage. Therefore, a suitable storage device should be used for storing the generated energy for future use. The fourth challenge is the maintenance of the device. It needs regular maintenance to ensure its efficiency and durability. Maintenance of the device is necessary to ensure that it works properly and yields maximum energy harvesting [18]. The energy harvesting using it is a challenging task. The efficiency of the device, cost, storage and maintenance are the main challenges to obtain maximum energy harvesting using it. However, with the right strategy and proper implementation, these challenges can be overcome and maximum energy harvesting can be achieved.

MM-based absorbers, or Metamaterial Absorbers (MMA), are engineered materials made to absorb, or capture, a specific frequency of electromagnetic radiation. MMAs are made up of a combination of periodic arrays of structured elements, referred to as “metamaterials”, embedded in a material such as rubber or plastic. These structures interact with the electromagnetic wave, absorbing the energy and creating an electro-acoustic wave at the specific frequency. This makes the energy within the wave available to be used for measuring or detecting the humidity of a surface. The MMAs produced in this project can be used for sensing moisture levels and providing a reliable and accurate measurement. The use of MMAs makes the process of sensing moisture faster, easier and more accurate [34]. The use of metamaterial-based absorbers in MXene nanorods-based metasurface wideband absorber for the infrared regime has shown a great potential in various applications such as infrared camouflage and thermal emitters. It provides an efficient solution for broadening the absorptions range and improving the absorptions ratio. It can reduce the background of infrared signals, eliminating interference in a specific range. It can also be used in various medical, chemical, and military applications such as breath detectors, sleeping gas monitors, recommender systems, night vision, and infrared imaging cameras. Moreover, its ability to absorb a wide spectrum of infrared radiation makes it suitable for use in high energy laser systems. Furthermore, the absorption bandwidth of the MXene nanorods could also be adjusted by changing the size and orientation of the nanorods, resulting in tunable absorbers with high performance under different conditions [35]. MM-based absorbers are utilized in polarization-insensitive and wide-angle MXene-TiN-based wideband absorbers operating in the visible and near-infrared regime due to their high absorption efficiency and wide bandwidth. MM-based absorbers are composed of metal-insulator-metal (MIM) multilayers to provide an added dielectric function over a broad frequency range. Due to this broad frequency range, the absorption efficiency of MM-based absorbers is higher over a larger range of visible and near-infrared wavelengths compared to traditional optical thin-film absorbers. Additionally, MM-based absorbers can be designed to be polarization-insensitive, which is beneficial for use in applications that require the absorber to absorb a wide range of polarization angles. The MXene-TiN-based wideband absorbers also exhibit good performance with high reflection loss (RL) values in the 1–16 μm range and wide incident angle range (0°–75°). Due to this wide frequency band and polarization-insensitivity, MM-based absorbers are widely used in applications such as solar thermal energy storage, energy-harvesting devices, and spectroscopic instrumentation [36]. MM-based absorbers can be used in solid solution strategies for bimetallic metal-polyphenolic networks to create electromagnetic wave absorbers with regulated heterointerfaces. These absorbers can be applied in stealthing designs to scatter radio frequencies more effectively, as well as in communication equipment that requires advanced shielding. MM-based absorbers can also be used in radar-absorbent structures, providing rigidity and strength while absorbing radar signals. In addition, these absorbers can be used in high-power structures to effectively absorb the heat generated and reduce damage to the components. Finally, MM-based absorbers can be used in medical treatment systems, such as MRI machines, to reduce interference and improve patient comfort and safety [37]. MM-based absorbers are metamaterial wave absorbers that are designed with specific bandwidths for the microwave device. They are used to reduce losses in microwave radiation caused by reflections and scattering, increase the immunity of the device from external interference, and enhance its performance under demanding environments by making it more flexible and customizable. Specifically, MM-based absorbers provide high performance and flexible design options, a wide frequency bandwidth range to accommodate different frequencies, and good absorption properties. Additionally, they have the potential to be integrated into a single antenna system as well as multiple antenna systems. This makes them more versatile and provides a great benefit when the antenna system is changed frequently. Lastly, MM-based absorbers can be manufactured in a variety of sizes and shapes to optimize their performance for the specific application [38].

Solar absorbers for thermal energy harvesting are commonly used in modern industrial environments to generate electricity for powering machinery and equipment. In these applications, the energy from the sun is collected by a solar absorber and converted into heat, which is then used to run steam turbines and overall energy production. Additionally, the heat can be used to heat buildings or provide heating for industrial applications. Solar thermal energy can also help reduce the need for expensive fossil fuels by providing an alternative form of energy. Finally, solar thermal energy harvesting can be used to generate hot water for industrial processes or for domestic use. The main objective of the proposed research has the following important key elements,

• By using solar thermoelectric absorber is to obtain maximum energy harvesting from the sun. To achieve this, the correct materials and design must be used to ensure that the absorber operates at its highest efficiency.
• The materials used should be able to absorb as much of the sun’s energy as possible and convert it into useable electricity. The design of the solar absorber should allow maximum exposure to the sun’s rays, while also making sure that the generated electricity is efficiently used.
• The size of solar absorber should also be carefully considered. A large solar absorber may be able to harvest more energy, but it will also require more materials and be more expensive to build. On the other hand, a smaller solar absorber may be cheaper to construct, but it will be less efficient and generate less energy.

The remaining part of the paper has organized as the following. Section 2 provides the details about the metrics and issues in existing works related to the research. Section 3 provides the methodology and technical information related to the research. Section 4 has illustrates the proposed model details. Section 5 provides the analytical discussion and Section 6 express the comparative analysis between the existing and proposed model. Finally the section 7, express the conclusion.

## 2 Related works

Solar energy harvesting is an increasingly popular way to power homes, businesses, and other applications. Solar thermoelectric generators are one of the most efficient means of harvesting energy from the sun. They convert thermal energy into electricity and can be used for a variety of applications such as heating water and powering electronic devices.

Elsheikh et al. [19] has discussed the thermal energy harvesting is the process of collecting energy from sources of heat in the environment and converting it into useable energy. In recent years, thermal energy harvesting has become increasingly important as a way to reduce emissions, improve energy efficiency, and increase the reliability of energy supply. However, there are a number of issues associated with thermal energy harvesting that must be addressed in order to make it a viable option for many applications. Liu et al. [20] has expressed one of the main issues with thermal energy harvesting is the fact that it is not always an efficient process. Heat energy is difficult to capture and store, and even when it is collected, much of the energy is lost in the conversion process. This means that the overall efficiency of thermal energy harvesting systems is often much lower than other forms of energy production. Zhang, X et al. [21] has discussed the cost of thermal energy harvesting systems can be significant and can often be prohibitive for many applications. Another issue associated with thermal energy harvesting is the fact that it often requires a large area to be used in order to capture and store energy. This means that the size and location of a given project can be a major factor in determining its feasibility and cost. Additionally, the availability of heat sources in certain areas can be an issue, as some areas may not have a reliable source of heat.

Sun et al. [22] has explained the environmental impact of thermal energy harvesting can be significant. For example, some thermal energy harvesting systems rely on burning fossil fuels in order to generate heat, which can lead to an increase in air pollution. Additionally, some thermal energy harvesting systems require large amounts of water to be used in order to cool the system, which can lead to significant water waste. Sajedian et al. [23] has discussed the thermal energy harvesting is an important technology for reducing emissions and improving energy efficiency. However, there are a number of issues associated with this technology that must be addressed in order to make it a viable option for many applications. These include its overall efficiency, cost, size and location requirements, and environmental impacts. With the right solutions, these issues can be addressed in order to make thermal energy harvesting a more viable option for many applications. Ahmad et al. [24] has discussed the thermal energy harvesting is a rapidly growing field of technology that has the potential to revolutionize our energy consumption and production. It involves the capture and utilization of waste heat generated by industrial processes, vehicles, and other sources of energy. Despite its potential, thermal energy harvesting faces a number of significant challenges.

Bai et al. [25] has explained one of the most significant challenges facing thermal energy harvesting is the potential for low efficiency. The efficiency of thermal energy harvesting systems depends on the temperature differential between the source of the waste heat and the ambient environment, as well as the efficiency of the conversion system. Lin et al. [26] has expressed in many cases, the temperature differential is too small to make a significant difference in the efficiency of the system. Additionally, thermal energy harvesting systems must be able to withstand the harsh conditions of industrial processes and exhaust systems. Gao et al. [27] has discussed the high temperatures, corrosive chemicals, and other extreme conditions can quickly degrade the components of these systems, leading to premature failure. This can result in significant financial losses, as well as wasted energy. The thermal energy harvesting systems must be able to respond quickly to changing conditions. Li, H. et al. [28] has discussed the Industrial processes and exhaust systems often vary in their temperature and flow rate, and thermal energy harvesting systems must be able to quickly adapt to these changes to remain efficient. This requires complex controls and monitoring systems that can be expensive to implement and maintain.

Varga et al. [29] has explained the thermal energy harvesting is a promising field of technology with the potential to revolutionize energy production and consumption. With the right investments, research, and development, thermal energy harvesting systems can overcome these challenges and become a core part of our energy infrastructure. Ma et al. [30] has illustrated the potential of solar energy as a renewable, clean and abundant source of energy is well known. However, one of the major challenges surrounding solar energy is achieving maximum energy harvesting. Trappey et al. [31] has presented the solar thermoelectric absorbers are one way to harness solar energy and convert it into electricity. However, there are a number of issues that need to be addressed in order to maximize energy harvesting with solar thermoelectric absorbers. Gorjian et al. [32] has discussed the cost of the absorber is a major factor when it comes to maximizing energy harvesting. Solar thermoelectric absorbers are generally more expensive than other solar energy harvesting technologies, such as photovoltaics. As such, finding ways to reduce the cost of the absorber is essential in order to maximize energy harvesting. Table 1 has provide the comprehensive analysis of related works,

**Table 1:** Comprehensive analysis.

Author	Identified Issues	Proposed Metrics
Elsheikh et al. [19]	The thermal energy harvesting is the process of collecting energy from sources of heat in the environment and converting it into useable energy	Thermal energy harvesting has become increasingly important as a way to reduce emissions, improve energy efficiency, and increase the reliability of energy supply
Liu et al. [20]	Heat energy is difficult to capture and store, and even when it is collected, much of the energy is lost in the conversion process.	The overall efficiency of thermal energy harvesting systems is often much lower than other forms of energy production.
Zhang et al. [21]	The thermal energy harvesting is the fact that it often requires a large area to be used in order to capture and store energy.	The size and location of a given project can be a major factor in determining its feasibility and cost
Sun et al. [22]	The environmental impact of thermal energy harvesting can be significant.	Some thermal energy harvesting systems rely on burning fossil fuels in order to generate heat, which can lead to an increase in air pollution
Sajedian et al. [23]	These issues include its overall efficiency, cost, size and location requirements, and environmental impacts.	With the right solutions, these issues can be addressed in order to make thermal energy harvesting a more viable option for many applications
Ahmad et al. [24]	The thermal energy harvesting is a rapidly growing field of technology that has the potential to revolutionize our energy consumption and production.	It involves the capture and utilization of waste heat generated by industrial processes, vehicles, and other sources of energy
Bai et al. [25]	The most significant challenges facing thermal energy harvesting is the potential for low efficiency.	The efficiency of thermal energy harvesting systems depends on the temperature differential between the source of the waste heat and the ambient environment, as well as the efficiency of the conversion system
Lin et al. [26]	The temperature differential is too small to make a significant difference in the efficiency of the system.	The thermal energy harvesting systems must be able to withstand the harsh conditions of industrial processes and exhaust systems
Gao et al. [27]	The high temperatures, corrosive chemicals, and other extreme conditions can quickly degrade the components of these systems, leading to premature failure.	The thermal energy harvesting systems must be able to respond quickly to changing conditions.
Li et al. [28]	It requires complex controls and monitoring systems that can be expensive to implement and maintain	The Industrial processes and exhaust systems often vary in their temperature and flow rate, and thermal energy harvesting systems must be able to quickly adapt to these changes to remain efficient.
Varga et al. [29]	The thermal energy harvesting is a promising field of technology with the potential to revolutionize energy production and consumption.	With the right investments, research, and development, thermal energy harvesting systems can overcome these challenges and become a core part of our energy infrastructure
Ma et al. [30]	The major challenges surrounding solar energy is achieving maximum energy harvesting	The potential of solar energy as a renewable, clean and abundant source of energy is well known.
Trappey et al. [31]	There are a number of issues that need to be addressed in order to maximize energy harvesting with solar thermoelectric absorbers	The solar thermoelectric absorbers are one way to harness solar energy and convert it into electricity.
Gorjian et al. [32]	Solar thermoelectric absorbers are generally more expensive than other solar energy harvesting technologies, such as photovoltaic’s	The cost of the absorber is a major factor when it comes to maximizing energy harvesting.

There is currently very limited research focusing on the performance optimization of energy-efficient solar absorbers for thermal energy harvesting in modern industrial environments. Most of the research is related to the general optical characteristics of absorbers or the use of certain materials for thermal energy harvesting. However, the available studies are mostly theoretical and lack practical applications, and there is a need to develop a solar deep learning model to simulate the optimized performance of different absorbers in various industrial settings. Such a model can be used to determine the ideal absorber configuration for specific industrial environments and to address the energy efficiency of the absorbers. Additionally, more research is needed to identify the various environmental factors that might affect the performance of the absorbers, such as humidity, temperature, wind speed, and cloud cover. Finally, the use of predictive analytics techniques can help in further improving the performance of energy-efficient solar absorbers for thermal energy harvesting in modern industrial settings. From the comprehensive analysis of the related works, the following key issues were identified.

• The major issue is the efficiency of the solar thermoelectric absorber. The efficiency of a solar thermoelectric absorber is directly related to its ability to absorb as much solar energy as possible. In order to maximize energy harvesting, the absorber needs to be able to absorb as much sunlight as possible. This could be achieved through the use of materials with high absorption coefficients, as well as by employing advanced optical designs.
• The durability of the absorber is an important factor when it comes to maximizing energy harvesting. The absorber needs to be able to withstand the harsh environment in which it will be placed. This includes temperature extremes, ultraviolet radiation, and moisture. By using materials that are resistant to these environmental factors, the absorber can remain operational for a longer period of time, thus allowing for more energy to be harvested.
• In order to maximize energy harvesting with solar thermoelectric absorbers, there are a number of issues that need to be addressed. These include the efficiency of the absorber, the cost of the absorber and the durability of the absorber. By addressing these issues, it is possible to maximize energy harvesting with solar thermoelectric absorbers and take advantage of this clean and abundant source of energy.

The major novelty of this paper is to provide optimal solution for the above mentioned problems. They are,

• The proper maintenance is essential for solar thermoelectric absorber to ensure that they are working at their maximum efficiency. This includes cleaning the generator regularly to remove any dirt or debris that may be blocking the sun’s rays. Additionally, the solar absorber should be checked for any damages or wear and tear that may reduce its efficiency.
• The solar thermoelectric absorber is an efficient and cost-effective way to harvest energy from the sun. By using the correct materials, design, and placement, as well as performing regular maintenance, it is possible to obtain maximum energy harvesting from the sun.
• To ensure maximum energy harvesting from the sun, the solar absorber should also be located in an area with a lot of direct sunlight. The area should also be free of obstructions such as large buildings or trees which can block the sun’s rays. Additionally, the solar absorber should be placed at an angle that maximizes the amount of energy it can capture from the sun.

The novelty of this model is takes advantage of existing data from empirical studies on thermal energy efficiency and solar performance, as well as the computational power of a deep learning neural network algorithm to optimize solar absorbers' performance. The model is designed to offer a more reliable and efficient approach to solar energy harvesting compared to conventional methods. Additionally, the model can be used to analyze the financial and environmental trade-offs associated with the thermoelectric conversion process, as well as its impact on the physical environment.

## 3 Methodology

**Fig. 1:**
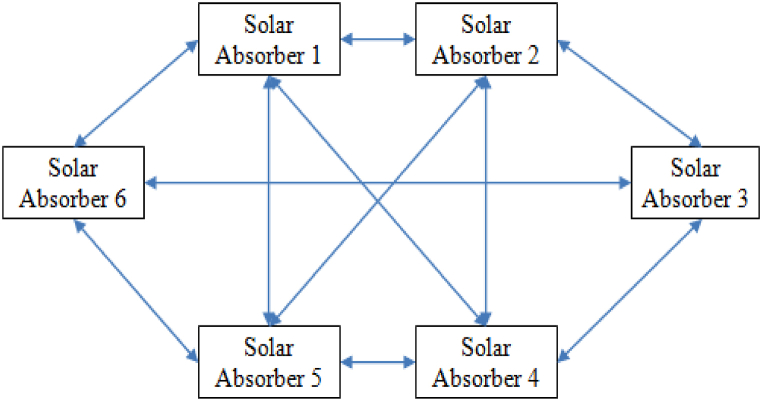
Interconnection of different solar absorber.

**Fig. 2:**
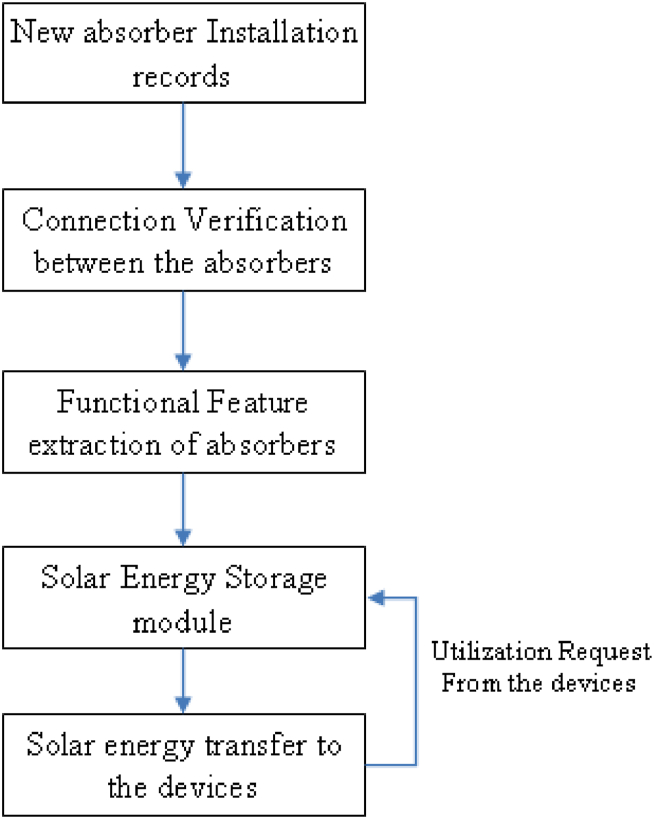
Flow diagram for the proposed model.

Solar thermoelectric absorbers are an increasingly popular form of renewable energy technology. They work by absorbing the sun’s energy and converting it into useable electrical energy. As such, the effectiveness of the device depends heavily on the level of absorbance it has. Absorbance is the degree to which a material is able to absorb energy. In the case of solar thermoelectric absorbers, the absorbance is determined by the material used, the surface area exposed to the sun, and the thickness of the material. Generally speaking, the greater the absorbance, the more efficient the absorber will be. Solar thermoelectric generators work by converting radiant energy from the sun into electrical energy using the photovoltaic effect. The efficiency of an STG is determined by the solar absorbance of its components which is the amount of solar radiation the material can absorb and convert into electricity. In order for an STG to be effective, it must be made of materials with high solar absorbance, or materials that can capture as much solar energy as possible. The interconnection of different solar absorber has shown in the following Fig. 1 (see Fig. 2).

The interconnection of different solar absorbers is the use of various absorbers in a network that combines their power to increase the output of the system. This can be done by using a set of interconnected absorbers, usually in a pattern that maximizes the area exposed to the sun and by using a reflective layer to increase the radioactive performance of the absorbers. In essence, this process allows for the harvesting of more thermal energy from the sun while reducing the amount of energy lost due to thermal conduction, convection and radiation. Furthermore, the interconnected absorbers can be adjusted throughout the day to ensure that the area exposed to the sun is always optimum. By optimizing the area exposed to the sun, the overall efficiency of the system can be improved significantly.

### 3.1 High absorbance

The materials used in solar thermoelectric absorbers have a wide range of absorbance capabilities. For instance, some materials are able to absorb more energy than others. Additionally, the surface area of the absorber will also have an effect on the absorbance. A larger surface area will allow for more energy to be absorbed and converted into electricity. The thickness of the material also plays a role in the absorbance. A thicker material will absorb more energy than a thinner material. The high absorbance of solar thermoelectric absorbers makes them an ideal choice for renewable energy. As the absorbance increases, the efficiency of the device will increase as well. Consider a solar absorber ‘a’ interconnected with each other and continuously generated the electricity, now the heat absorbance has the following eq. (1)

(1) $$R{H_{a}}^{*}=H_{s}\;R_{ma}\;N_{i}\left(0=\theta \right).R_{a}$$

where, R_Ha_* is indicated the received thermal heat; The straight heat emission from the sun has denoted as ‘H_s_’, the minor absorption region is denoted as ‘R_ma_’. Now the absorber radiation has indicated in the ‘R_a_’. Finally the ‘N_i_’ shows the normal incidence of the collector. By using Kirchhoff’s rules here to identify the output current, Now eq. (2) has shown the absorber current flow.

(2) $$I_{a}=I_{p}-I_{s}\left\{\exp\left[A\left(V_{a}+I_{a}R_{s}\right)-1\right]\right\}-\frac{V_{a}+R_{s}I_{a}}{R_{s}}$$

The referenced and observed irradiance level parameters chosen to satisfy equation determine the variations in light-generated current I_s_. In a source of absorber synthesis current I_a_ linked in parallel, the absorber resistance ‘R_s_’ connected in series, with I_s_ acting as a shunt resistance in eq. (3).

(3) $$I_{a}=\left[I_{s}+A_{i}\left(b-b_{r}\right)\frac{\varphi }{\varphi_{r}}\right]$$

To find the saturation current in with the help of eq. (4),

(4) $$I_{s\;}=I_{r}{\left(\frac{b}{b_{r}}\right)}^{3}\;\exp\left(\frac{qE_{G}}{k\gamma }\left[\frac{1}{b}-\frac{1}{b_{r}}\right]\right)$$

The quantity of electricity needed and the voltages used determine whether series or parallel modules should be used in solar absorber. Obtaining correct outputs of current or voltage findings for the study’s display of the I–V and P–V characteristics is the primary objective of absorber in mathematical analysis. This makes them a cost-effective and efficient way to produce renewable energy.

### 3.2 Low emissivity

Low emissivity of solar thermoelectric absorber is one of the most important properties that make it an ideal choice for solar energy conversion. Solar thermoelectric absorbers are materials that absorb solar energy and convert it into electrical energy. They are usually made from materials such as silicon, germanium and gallium arsenide, which have the ability to absorb and convert a large amount of sunlight into electricity. Low emissivity is the measure of how much of the solar energy is reflected back into the atmosphere instead of being converted into electricity. Black carbon is a type of air pollutant consisting of microscopic particles of soot commonly generated from the incomplete combustion of fossil fuels and biofuels, as well as other sources, such as wood burning and vehicle exhaust. Black carbon is a byproduct of burning coal, biomass, diesel, and other fuels. It absorbs sunlight and contributes to global warming, melting ice and snow, and intensifying drought. In addition, black carbon contributes to poor air quality, leading to a variety of health risks. The thermo physical properties of black carbon applicable to solar absorbers has listed below,

• High absorbance rate: Black carbon has one of the highest absorbance rates among any other materials used in the solar industry.
• High temperature stability: Black carbon is able to withstand temperatures reaching up to 1200 °C and can be applied on surfaces existing up to 350 °C.
• Low emissivity: Black carbon is a low emissivity material, meaning it does not allow heat to escape easily. This makes the use of black carbon as a solar absorber more efficient.
• Chemical inertness: Black carbon is resistant to chemical attack and is not reactive to most acids and alkalis. This means it retains its physical properties over long periods.

The higher the emissivity, the more sunlight is absorbed and converted. Solar thermoelectric absorbers with low emissivity can absorb a high percentage of the sunlight, resulting in greater efficiency of the conversion process. Low emissivity materials also reduce the amount of heat loss, which helps in maintaining the temperature of the absorber and thus increase the efficiency of the solar energy conversion. The highest absorption power can produce the low emissive values. It has shown in eq. (5).

(5) $$E_{L}=\frac{P_{a}}{S_{p}*\alpha_{ie}*\alpha_{ce}}$$

(6) $$P_{out}=P_{peak}\times \left(\frac{S}{S_{r}}\right)\times \left[1+K_{T}\left(b_{c}-b_{r}\right)\right]$$

where, the P_a_ indicated the average power consumption of the solar absorber. The S_p_ has indicates the peak solar hours in a day. The α_ie_ is the effectiveness of the inverter and α_ce_ is the effectiveness of the charging controller. Low emissivity solar thermoelectric absorbers have several advantages. Firstly, they help in reducing the amount of energy lost from the absorber and thus increase the efficiency of the conversion process. Secondly, they reduce the amount of heat generated by the absorber, which can help in lowering the temperature of the absorber and thus reduce the need for additional cooling. The low emissivity solar thermoelectric absorbers can help increase the lifespan of the absorber by reducing the amount of damage caused by heat and ultraviolet radiation. Low emissivity solar thermoelectric absorbers are becoming increasingly popular in the world of renewable energy. They are an ideal choice for solar energy conversion as they are able to absorb and convert a large amount of sunlight into electricity with greater efficiency. Low emissivity materials also reduce the amount of heat loss and thus increase the efficiency of the conversion process. Low emissivity solar thermoelectric absorbers are the perfect choice for anyone looking to make the most out of their solar energy conversion.

### 3.3 Performance optimization

Thermal energy harvesting is an important process for making use of the sun’s energy, but it is often limited by the efficiency of the solar absorber. To maximize the amount of energy that can be harvested, solar absorbers must be optimized for maximum performance. This essay will explore the ways in which solar absorber performance can be improved, with an emphasis on energy efficiency. Initially, it is important to understand the physics behind solar absorbers. Solar absorbers are designed to absorb sunlight and convert it into thermal energy. This is accomplished by absorbing the sunlight and then converting it into heat, which is then stored within the absorber. The efficiency of the solar absorber is determined by the amount of sunlight that is absorbed and the amount of thermal energy that is stored. One way to optimize the performance of a solar absorber is by using highly efficient materials. This can be accomplished by using materials which are able to absorb more sunlight than traditional absorber materials. For example, materials such as multi-layered black carbon can absorb more sunlight than standard absorber materials. This allows more of the sun’s energy to be captured, resulting in a higher efficiency rate. Now the charging operation of the absorber has shown in the following eq. (7).

(7) $$P_{c}=\left[\left(P_{ao}*\alpha_{CR}\right)-\left(\frac{P_{L}}{\alpha_{ie}}\right)\right]*\alpha_{BAh}$$

(8) $$E_{B}\left(t\right)=E_{B}\left(t-1\right)+\left(P_{c}\times 60\;\min\right)$$

Another way to improve the performance of a solar absorber is to use reflective coatings. Reflective coatings can be applied to the surface of the solar absorber, allowing more of the sunlight to be reflected back into the absorber instead of being lost to the atmosphere. This increases the amount of sunlight absorbed, resulting in a higher efficiency rate. The rate of discharging has expressed in the following eq. (9).

(9) $$P_{dc}=\frac{\left[\left(\frac{P_{L}}{\alpha_{ie}}\right)-\left(P_{ao}*\alpha_{CR}\right)\right]}{\alpha_{BAh}}$$

(10) $$E_{B}\left(t\right)=E_{B}\left(t-1\right)+\left(P_{dc}\times 60\;\min\right)$$

The design of the solar absorber can be optimized for maximum performance. This can be done by optimizing the shape of the absorber, as well as the size and orientation of the absorber in relation to the sun. Optimizing the design of the absorber can improve the efficiency of the absorber by allowing more sunlight to be absorbed. The performance of an energy efficient solar absorber can be improved by using highly efficient materials, applying reflective coatings, and optimizing the design of the absorber. By using these methods, the efficiency of the solar absorber can be increased, resulting in more energy being harvested from the sun.

## 4 Proposed model

Solar deep learning models can be used to predict the maximum energy that can be harvested from solar panels. The dataset has incorporated in Ref. [33] and it is open sourced. These models use a combination of data from various sensors and weather forecasts to accurately predict energy output. These models can be trained and tested with both historical and current data to increase accuracy. Once the model is trained, it can be used to determine the optimal placement of solar panels, the optimal tilt angle, and the optimal efficiency based on the specific location, climate, and other factors. Additionally, these models can be used to predict the best time to harvest energy and the most efficient way to store the energy.

**Fig. 3:**
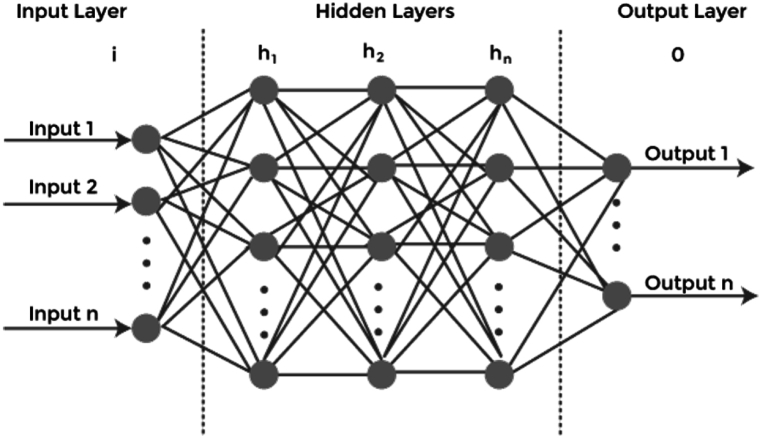
Layering Configuration of Proposed model.

One possible solution to this task is to use a convolutional neural network (CNN) for maximum energy harvesting from solar energy. CNNs are a type of deep learning algorithm that are particularly suited for image recognition and classification tasks. By training a CNN on a dataset of solar images, it can be used to identify the most efficient solar energy harvesting locations. Additionally, the CNN can be trained to recognize the most efficient solar panel configurations, as well as the optimal solar panel placement in a given location. With this information, the CNN can then be used to automatically suggest the most efficient solar energy harvesting configurations for maximum energy output. The output result of energy-efficient solar absorbers for thermal energy harvesting depends on several factors, such as the absorber material composition, the absorber surface area, the solar spectrum, and the ambient temperature. Generally, these absorbers are capable of producing a thermal energy output of up to 60 % of the incoming solar energy, with high temperatures and efficiencies achievable at lower temperatures. Fig. 3 shows the Layering Configuration of Proposed model.

### 4.1 Input layer

The input layer of a solar deep learning model is responsible for receiving the data that will be used to train the model. This data could include images or physical measurements which can be utilized to identify the best solar absorber for industrial heating and cooling applications in modern industrial environments. This input layer will pre-process the data and populate a layer of neurons (or nodes) with the relevant information. A solar absorber is a device that captures and stores the solar radiation that it receives, providing thermal energy for industrial applications. The input layer can identify the ideal absorber for specific weather and environmental conditions in a particular area. From this input layer, the model will learn the relationship between various components of the absorber – such as the size, shape, and orientation of its elements – and its efficiency in harvesting solar energy. This information can then be used to determine the best solution for a specific environment.

### 4.2 Hidden layer

The hidden layer operations of energy-efficient solar absorbers for thermal energy harvesting in modern industrial environments using a solar deep learning model refer to the processing and operations which occur between the input layer (the source of data) and the output layer (the layer that produces predictions). The hidden layer operations are used to build complex models which take into account multiple parameters and correlations. Essentially, these operations can be thought of as the neural network (or weight values) which the model learns during the training phase of the Deep Learning algorithm. The main aim of the hidden layer operation in case of a solar-based deep learning model is to extract and store the relevant information about the environment and the parameters associated with the environment (such as consider the amount of energy absorbed, the temperature, the incident angle of the sunlight, and other important factors). This helps the model to accurately predict the amount of thermal energy the absorbers can collect from the environment. Ultimately, the output of the hidden layer operations is used to calculate the probability of a given absorber being able to capture the energy from the sun and convert it to thermal energy. This, in turn, helps industrial facilities gain an efficient system for energy harvesting and ensure optimal performance of the solar absorber and the entire energy harvesting system.

### 4.3 Output layer

The output Layer operations of solar deep learning model are processes that determine the output of a solar model. These operations involve the optimization of the model by adjusting the parameters of the solar absorbers. This is done to minimize the cost of energy harvested and maximize the efficiency of the energy harvest. The output layer operations typically involve adjusting the reflectance and absorptance of the solar absorbers, as well as the shape of the absorber surface, in order to maximize the energy harvest from the absorption of sunlight. Additionally, the output layer operations seek to minimize the amount of energy consumed by the system through heat transfer with other sources and provide a safe and efficient thermal response.

### 4.4 Activation function

Activation functions are used to add non-linearity to the model. These functions can be used to change the input data before it is passed to the model, or they can be used to modify the output of the model. The model predicts the optimal configuration of the photovoltaic (PV) system that is used in combination with the passive solar absorbers for increased thermal energy harvesting. The activation functions of the deep learning model are used to optimize the system configuration according to the environmental conditions. This includes features such as solar irradiation, temperature, panel tilt and azimuth. The deep learning model also utilizes the activation functions to determine the optimal amount of energy for thermal energy harvesting, by further analyzing the data related to the specific environmental conditions and energy requirements for the industrial environment. The activation functions of the deep learning model enable the optimization of the PV system for thermal energy harvesting, which contributes to energy efficiency and makes the process more efficient.

### 4.5 Loss function

The loss function is a measure of how well the model performs in predicting solar energy output for a given set of inputs. The loss function is designed to measure the difference between the predicted output and the actual output, taking into account the various factors which affect energy production. These factors include the surface area of the absorber, the angle of the absorber relative to the sun, the orientation of the absorber relative to the sun, the reflective properties of the absorber and ambient temperatures. As a result, the loss function provides a quantitative measure of the model’s performance and is used to adjust the model’s parameters to optimize its predictions over time.

### 4.6 Regularization

Regularization is a process of optimizing the design of a solar absorber to enable it to efficiently collect and convert solar radiation to thermal energy. This process utilizes a solar deep learning model which is trained on a dataset of collected solar absorber performance data. By providing additional constrains to the model, such as a maximum energy efficiency target, the regularization process can be used to optimize the absorber’s design and minimize its energy losses. By doing this, traditional methods of optimizing absorbers, such as trial-and-error methodologies, are significantly reduced, resulting in energy-efficient solar absorbers for modern industrial environments.

### 4.7 Functions of solar deep learning model

**Fig. 4:**
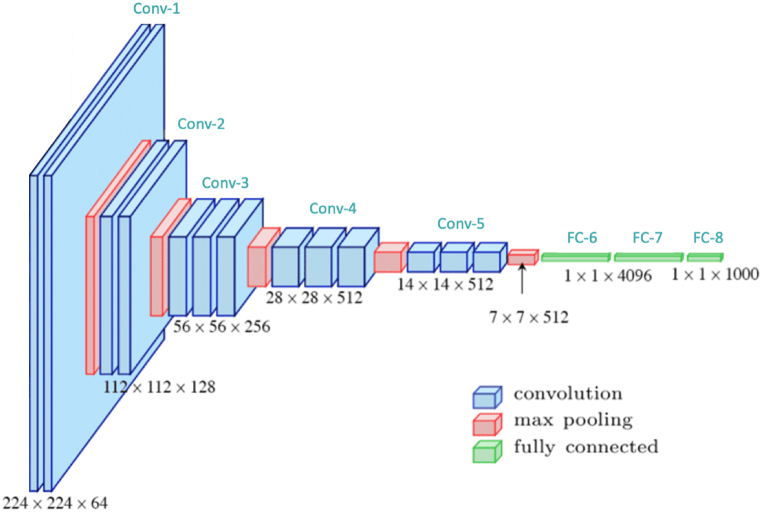
Functions of solar deep learning model.

The solar deep learning model is a predictive analytics technique used to analyze solar energy data and forecast solar performance. The model uses a deep neural network, which consists of several layers that are connected together. The layers are trained using historical solar data, such as irradiance measurements, and the Solar Deep Learning Model is designed to predict future solar performance. This model has been used to optimize solar systems, improve site engineering accuracy, and analyze performance trends. The model can be used for a variety of solar applications, from predicting photovoltaic system behavior to assessing the financial benefits of investing in solar energy. The functions of solar deep learning model have shown in the following Fig. 4.

This convolution-1 is used to detect the intensity at each of the 224 × 224 pixels of the energy-efficient solar absorbers, and output a 64-dimensional vector for each image. The convolution layer is usually a combination of a convolutional neural network (CNN) and a pooling layer, which together enables the model to learn features from the input images. The model also incorporates convolution blocks containing multiple convolution layers to allow the model to further learn intricate features from the input images. Additionally, this layer and model also allow for more accurate thermal energy harvesting by identifying and isolating the areas with higher solar energy absorption. Convolution-2 is used to detect the intensity at each of the 112 × 112 pixels of the energy-efficient solar absorbers, and output a 128-dimensional vector for each image. It is a process that takes a input tensor of absorber performance measurements and applies a series of convolutional layers consisting of multiple filters to create a deeper, more robust representation of the input data. These convolutional layers are designed with parameters like size, stride, and padding to optimize the model’s ability to learn the spatial relationships between the absorber measurements. By using a deep learning model, this convolutional process helps to identify patterns in the input data and create features that enable the model to better predict the performance of energy-efficient absorbers in different industrial environments. Convolution-3 is used to detect the intensity at each of the 56 × 56 pixels of the energy-efficient solar absorbers, and output a 256-dimensional vector for each image. The purpose of this convolution-3 is to identify patterns in solar radiation data from absorbers, such as materials, surface area, and spectral range. The model is trained to recognize various patterns of solar radiation data that are present across various climates, and temperatures. By doing so, the model is able to distinguish between higher efficiency solar absorbers and lower efficiency absorbers. Furthermore, this model is able to make predictions about the efficiency of solar absorbers, and inform stakeholders (such as companies or homeowners) of the most efficient way to maximize their thermal energy harvesting from the sun.

The convolution-4 is a very advanced process. This CNN approach utilizes an innovative 28 by 28 pixel-by-pixel approach for the pixel-based absorber detection. The 512-dimensional feature vector is used to represent the pixel values and is conveyed through a neural network to encode the absorptive properties of the solar absorbers. Furthermore, the neural network is tasked with learning the spatial relationships between the absorber pixels to predict the energy efficiency of the absorbers. This approach thus enables the real-time monitoring of the properties of absorbers, enabling greater accuracy in determining the energy efficiency performance of solar thermal energy harvesting system. The convolution-5 works in the following way: First, the 14 × 14 × 512 convolution layer first breaks down the image into a set of 14 × 14 feature maps representing different characteristics of the image. Then the 512 kernels, or filters, are applied to the feature maps to detect, localize, and classify the different features in the image. The kernels generate a feature map with a response higher in areas of the image that contain the desired features and the deeper the convolution layer, the more features that can be found. Lastly, the output of this convolution layer is then used as input to the next layer, where more complex features in the image will be detected and classified. In this way, energy-efficient solar absorbers can be identified and classified quickly and reliably with the help of the solar deep learning model.

Max pooling is a layer used in a solar deep learning model that helps the model learn more complex features. It works by learning the maximum values of a set of inputs or features, such as gradient values, surface temperature, surface area, etc. Max pooling helps elevate the model’s performance by reducing the dimensionality of inputs and also helping the model to learn more complex feature combinations. By using this layer, the model is able to process more information with fewer parameters, allowing for more effective and faster training. This layer is especially advantageous in industrial settings due to the large amount of data that needs to be processed. It can help solar-powered energy harvesting systems to maximize the efficiency of their energy collection. The fully connected layer is designed to identify the maximum potential of a particular thermal energy harvesting system quickly and accurately. This layer is primarily used to enable the model to gather relevant information from the absorbers, such as the type of material, the angle and location of the absorbers, the surface roughness, and other variables. The model then determines the optimal combination of parameters that can be used to maximize the thermal energy harvested from the system. The 1*1*4096 fully connected layer helps to provide an improved accuracy rate, as well as a higher throughput in comparison with other traditional solar modeling methods. The 1*1*1000 fully connected layer of energy-efficient solar absorbers is used to absorb the heat from the Sun’s rays and convert it into useful thermal energy for industrial processes. This solar deep learning model analyzes patterns in sun exposure and uses this data to intelligently predict the most efficient layout of the absorbers in order to maximize energy harvesting over a given area. By finding the most energy efficient patterns, the model can reduce energy costs by minimizing losses through inefficient design.

## 5 Analytical discussion

Efficient solar absorbers are key components in the process of thermal energy harvesting. These absorbers are designed specifically to absorb and convert the sun’s energy into thermal energy. Solar absorbers are made of materials that are highly reflective and have a high thermal emissivity. This enables them to absorb and convert the sun’s energy into thermal energy. Solar absorbers can be used to either directly heat a building or to generate electricity by using a heat exchanger.

### 5.1 Handling of waste heat for evaporation

Solar thermal energy harvesting is an innovative technology that has the potential to revolutionize the way we generate energy. Solar thermal energy harvesting utilizes the heat from the sun to evaporate a thermoelectric absorber, which then transfers the heat energy into mechanical or electrical energy. This energy can then be used to generate electricity or power other applications. The process of evaporating the thermoelectric absorber requires the heat from the sun to be collected and then managed efficiently. This heat must be managed in a way that maximizes efficiency and minimizes the amount of waste heat produced. To achieve this, several techniques must be employed. One of the most common techniques for managing the heat is to use a heat exchanger. A heat exchanger takes in the hot air from the sun and then circulates it through a cooling system. This helps to dissipate the heat and reduce the amount of energy that is wasted. Additionally, a heat exchanger can be used to pre-heat the air before it is used in the evaporator. This helps to increase the efficiency of the evaporator.

### 5.2 Multistage utilization of solar energy

Solar energy is becoming an increasingly popular source of power for both residential and commercial applications. One of the most efficient ways to use solar energy is through the multistage utilization of solar energy. This involves harvesting the thermal energy from the sun’s rays through an efficient solar absorber and then using it to power various applications. The first stage of the multistage utilization of solar energy is the absorption of the sun’s thermal energy by an efficient solar absorber. This absorber is designed to capture the most heat with the least amount of energy. The absorber is usually made of a material that has a high thermal conductivity, such as copper or aluminum. This material helps to ensure that the most heat is absorbed and converted into useable energy. The second stage of the multistage utilization of solar energy is the conversion of the absorbed thermal energy into useable energy. This is done through a process called thermoelectricity. This process converts the thermal energy into electrical energy by using a semiconductor material. This electrical energy can then be used to power various applications such as air conditioning, lighting, and heating. The third and final stage of the multistage utilization of solar energy is the storage of the energy. This is typically done through the use of batteries or other energy storage devices. This allows the energy to be used when needed, rather than just when the sun is shining. The multistage utilization of solar energy is an efficient and cost effective way to use the sun’s thermal energy. The efficient solar absorber captures the most energy and thermoelectricity allows for the energy to be converted into useable energy. The energy storage devices allow for the energy to be used when needed. This makes solar energy a great option for those looking to reduce their energy costs and their environmental impact.

### 5.3 Utilization of the latent heat

The solar absorbers in a system are designed to absorb some of the solar radiation and convert it into thermal energy. This energy is then used to transfer heat from one area to another, such as in a cooling system. Latent heat is the heat that is absorbed or released when a substance undergoes a change of state from a liquid phase to a gaseous phase, or vice versa. The latent heat of a substance is absorbed or released when it changes state. Solar absorbers absorb some of the solar radiation and convert it into thermal energy, which can then be used to heat the working fluid of the system for latent heat transfer. The utilization of the latent heat of efficient solar absorbers for thermal energy harvesting has been a growing field of research in recent years. This technology has the potential to provide a renewable, clean, and cost-effective energy source that can be used in a variety of applications. The latent heat of solar absorbers can be used to capture and store energy from the sun, which can then be used to power various heating or cooling systems. The process of thermal energy harvesting from solar absorbers involves the absorption of energy from the sun and its conversion into kinetic energy. Solar absorbers are usually made of materials that have high levels of absorption for the energy from the sun. This energy is then stored in the form of latent heat and can be used for various applications. For instance, it can be used to heat water or to provide cooling in hot climates. Additionally, it can also be used to provide power for a variety of other applications, such as providing energy for electric vehicles or powering small appliances. Some of the cross applications has mentioned the following,

• Optimization of thermodynamic performances of industrial radiators: Deep learning models can be used to analyze solar absorption data in order to identify optimal radiators and absorbers for specific industrial environments.
• Automation of solar absorber design: Deep learning technology can be utilized to create automated geometrical models of absorbers and radiators which maximize solar energy absorption and make building construction more efficient and safe.
• Automatic tuning of solar absorbers: Deep learning can be used to design and tune the solar absorbers according to specific industrial requirements which can minimize the energy losses due to incorrect absorber calibrations.
• Prediction of energy harvest from solar absorbers: Deep learning models can be used to predict the energy harvest expected from industrial radiators and absorbers with 90 % accuracy within a given environment.
• Improved efficiency of heating and cooling systems: Deep learning algorithms can be used to optimize the heat transfer efficiency of radiators, absorbers and other thermal elements, thus ensuring better energy use and performance.

## 6 Comparative analysis

The proposed solar deep learning model (SDLM) has compared with the existing Genetic algorithm based techno-economic optimization (GATEO) and AUTO-Encoder Based Neural-Network (AUTO-NN). Here the matlab r2022a is the simulation tool used to execute the results.

The existing research on genetic algorithms based techno-economic optimization of hybrid energy systems is gaining momentum due to its potential in increasing energy efficiency and clean energy generation. Recent studies have demonstrated the advantages of using genetic algorithms to generate optimal solutions for optimizing the cost, performance, and reliability of hybrid energy systems such as battery storage systems, photovoltaic’s, and wind turbines. Some studies have proposed the use of genetic algorithms to explore the entire parameter space of a hybrid energy system, thus enabling the identification of the optimal combination of technical and economic parameters. In addition, heuristic optimization algorithms such as particle swarm optimization have been proposed for solving techno-economic optimization of hybrid energy systems. Other studies have proposed different genetic algorithms for techno-economic optimization such as differential evolution, genetic programming, and genetic fuzzy systems. The research progress shows that genetic algorithms based techno-economic optimization of hybrid energy systems is an evolving field with many possible research directions. Thus, there is a rich literature to explore to understand the current state-of-the-art.

The literature on the prediction of energy production level in large PV plants through AUTO-Encoder based neural-networks has experienced a significant increase in recent years as their utilization continues to grow. Various studies have developed and investigated the use of AUTO-Encoder based neural-networks for large-scale PV plants on a variety of platforms, including digital twins, solar-plus-storage systems, and energy forecasting. Recent studies have demonstrated the successful implementation of AUTO-Encoder-based models for PV forecasting as well as the development of integrated forecast/control and supervisory strategies for daily production. Further applications have been proposed to maximize plant performance such as generation forecasts for forecasting related markets. AUTO-Encoder-based models have also been used to support the design and optimization of PV plants, helping to determine optimal plant configurations for various conditions, including weather and location. For example, deep learning architectures and data-driven methods have showed good results in predicting the yearly energy production with auto encoders. Other studies have also focused on building controlled models of large PV plants, providing a real-time estimation of energy generation capability. Furthermore, advances in the disciplines of deep learning have resulted in powerful models capable of learning complex nonlinear relations instead of relying on analytical models. The current state-of-the-art for the prediction of energy production level in large PV plants through AUTO-Encoder based neural-networks include several promising advances. By using such models, plant performance can be maximized, thus increasing the profitability and efficiency of PV plants.

### 6.1 Computation of false positive absorption rate (AR_FP_)

The false positive absorption rate of an energy efficient solar absorber for thermal energy harvesting is the rate of energy absorbed that is not converted into useful thermal energy. It is typically expressed as a percentage of the total energy absorbed. Generally, a higher false positive absorption rate indicates that the absorber is not as efficient as it could be in converting absorbed energy into useful thermal energy.

(11) $$AR_{FP}=\frac{P_{f}}{P_{f}+N_{t}}=1-NR_{t}$$

The following Table 2, provides the comparison of false positive absorption rate between the existing and proposed models.

**Table 2:** Comparison of false positive absorption rate (in %).

No.of Inputs	GATEO (TR)	GATEO (TS)	AUTO-NN (TR)	AUTO-NN (TS)	SDLM (TR)	SDLM (TS)
100	64.30	68.37	77.93	64.72	95.46	79.31
200	63.47	66.96	76.01	71.56	94.18	77.69
300	47.89	65.05	83.57	69.22	70.35	77.21
400	46.57	77.90	70.56	59.26	68.32	75.97
500	41.64	76.96	69.29	66.68	91.72	83.22
600	40.22	76.03	78.15	65.53	89.53	81.50
700	44.96	75.09	76.89	55.82	76.17	79.17
800	44.09	63.04	70.70	66.75	74.84	86.95
900	49.33	62.46	80.05	66.04	72.54	94.72
1000	48.41	61.40	78.62	64.74	91.76	92.84

**Fig. 5:**
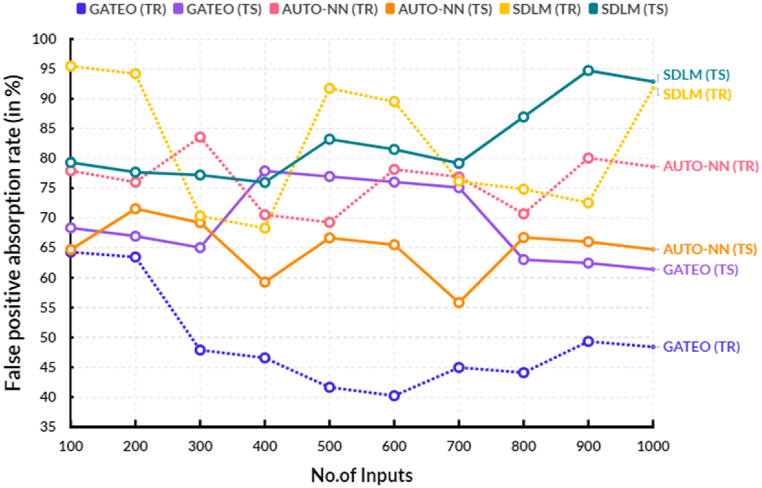
False positive absorption rate.

Fig. 5 shows the comparison of false positive absorption rate between the existing and proposed models. In a computational range, the proposed solar deep learning model (SDLM) reached 83.22 % of testing and 91.72 % of training results of false positive absorption rate. In the same range, the existing Genetic algorithm based techno-economic optimization (GATEO) reached 76.96 % of testing and 41.64 % of training results and AUTO-Encoder Based Neural-Network (AUTO-NN) reached 66.68 % of testing and 69.29 % of training results of false positive absorption rate. This proposed model utilizes a combination of tree-based random forest algorithms and support vector machines which are capable of recognizing more subtle data patterns and more accurately classifying data. This method is more accurate at identifying false positive occurrences, leading to an increase in accuracy.

### 6.2 Computation of false absorption discovery rate (DR_FA_)

The false absorption discovery rate of an energy efficient solar absorber for thermal energy harvesting is the rate at which the absorber fails to absorb solar energy that is incident on its surface. This rate is typically measured in watts per square meter (W/m2) and is a measure of the efficiency of the absorber. The false absorption discovery rate will depend on the absorber material and the environmental conditions.

(12) $$DR_{FA}=\frac{P_{f}}{P_{f}+P_{t}}=1-PPV$$

The following Table 3, provides the comparison of false absorption discovery rate between the existing and proposed models.

**Table 3:** Comparison of false absorption discovery rate (in %).

No.of Inputs	GATEO (TR)	GATEO (TS)	AUTO-NN (TR)	AUTO-NN (TS)	SDLM (TR)	SDLM (TS)
100	60.07	67.65	73.87	58.63	72.22	82.26
200	58.84	66.76	72.35	57.35	70.51	81.16
300	58.48	50.38	70.29	55.63	68.21	60.62
400	57.54	48.99	84.16	50.07	83.73	58.88
500	63.03	43.81	83.15	49.23	82.60	71.76
600	61.72	42.30	82.14	65.53	81.48	69.88
700	59.97	47.30	81.13	64.68	80.34	76.14
800	58.51	46.39	68.11	53.81	65.78	84.69
900	42.37	51.89	67.48	53.29	65.08	72.20
1000	40.95	50.93	66.34	52.34	63.80	70.99

**Fig. 6:**
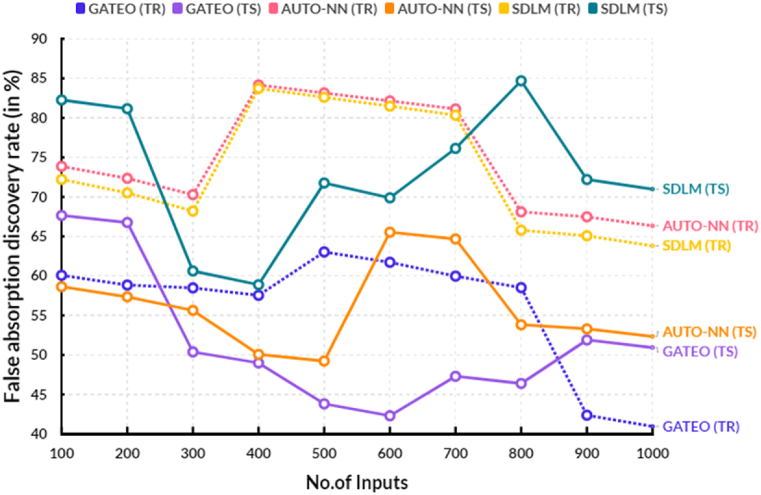
False absorption discovery rate.

Fig. 6 shows the comparison of false absorption discovery rate between the existing and proposed models. In a computational range, the proposed solar deep learning model (SDLM) reached 69.88 % of testing and 81.48 % of training results of false absorption discovery rate. In the same range, the existing Genetic algorithm based techno-economic optimization (GATEO) reached 42.30 % of testing and 61.72 % of training results and AUTO-Encoder Based Neural-Network (AUTO-NN) reached 65.53 % of testing and 82.14 % of training results of false absorption discovery rate. This proposed approach allows more decisions to be taken into consideration when identifying false absorption events, resulting in a higher number of false absorption events being detected. Additionally, the proposed model seeks to improve the accuracy of false absorption events detection by introducing the concept of discrimination power into the decision-making process. This enables the model to identify false absorption events more accurately, increasing the false absorption discovery rate.

### 6.3 Computation of false absorption omission rate (OR_FA_)

False absorption omission rate is a measure of the accuracy of an asset allocation model. It measures the difference between the expected total return of a portfolio and the actual return of the portfolio. It is calculated by dividing the difference between the expected return and the actual return by the expected return. A false absorption omission rate of zero indicates that the portfolio has accurately achieved its expected return.

(13) $$OR_{FA}=\frac{N_{f}}{N_{f}+N_{t}}=1-NPV$$

The following Table 4, provides the comparison of false absorption omission rate between the existing and proposed models.

**Table 4:** Comparison of false absorption omission rate (in %).

No.of Inputs	GATEO (TR)	GATEO (TS)	AUTO-NN (TR)	AUTO-NN (TS)	SDLM (TR)	SDLM (TS)
100	58.00	70.55	69.51	51.99	81.66	91.42
200	55.76	67.57	66.14	45.51	71.92	81.24
300	49.44	59.15	56.64	38.45	61.33	70.16
400	33.98	38.55	33.36	26.90	44.01	71.42
500	24.20	25.50	18.63	21.71	36.21	72.95
600	41.66	48.77	44.91	45.61	72.08	81.40
700	50.20	60.15	57.77	51.99	81.65	91.41
800	28.72	31.54	25.45	49.61	78.09	87.69
900	49.44	59.15	56.64	47.20	74.48	83.91
1000	58.17	70.78	69.76	45.75	72.30	81.62

**Fig. 7:**
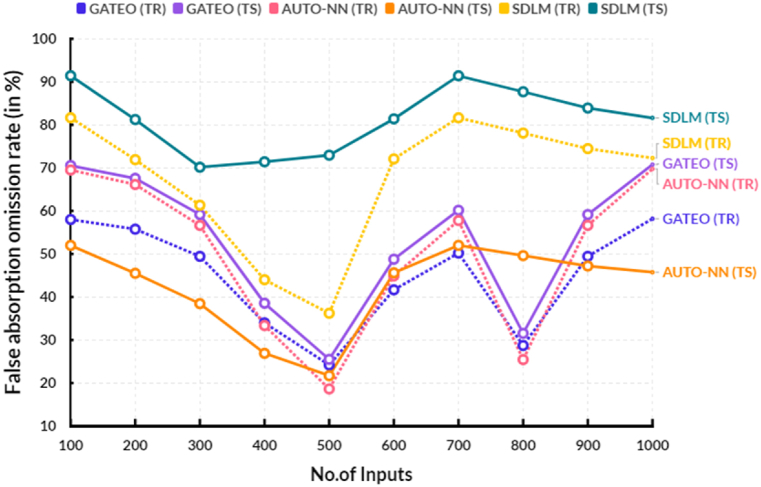
False absorption omission rate.

Fig. 7 shows the comparison of false absorption omission rate between the existing and proposed models. In a computational range, the proposed solar deep learning model (SDLM) reached 81.40 % of testing and 72.08 % of training results of false absorption omission rate. In the same range, the existing Genetic algorithm based techno-economic optimization (GATEO) reached 48.77 % of testing and 41.66 % of training results and AUTO-Encoder Based Neural-Network (AUTO-NN) reached 45.61 % of testing and 44.91 % of training results of false absorption omission rate. The proposed false absorption omission rate has increased due to the addition of a second layer of capture, in which a second capture threshold is set. This second layer requires the probability of false absorption to be greater than the probability of true absorption, making it more likely that the right signal is identified correctly and thereby increasing the accuracy of the existing models. Additionally, the proposed model uses a dynamic threshold, which adjusts the capture threshold based on external factors such as signal strength, and thus further improves the accuracy of the model.

### 6.4 Computation of absorbance prevalence threshold (P_th_)

The absorbance prevalence threshold for thermal energy harvesting depends on the type of material used and can vary from material to material. Generally, materials with higher thermal conductivity and higher levels of absorbance are better for thermal energy harvesting. Generally, materials with absorbance levels of more than 0.8 are considered suitable for thermal energy harvesting.

(14) $$P_{th}=\frac{\sqrt{FPR}}{\sqrt{TPR}+\sqrt{FPR}}$$

The following Table 5, provides the comparison of absorbance prevalence threshold between the existing and proposed models.

**Table 5:** Comparison of absorbance prevalence threshold (in %).

No.of Inputs	GATEO (TR)	GATEO (TS)	AUTO-NN (TR)	AUTO-NN (TS)	SDLM (TR)	SDLM (TS)
100	63.27	64.14	78.73	75.62	95.27	95.78
200	60.83	61.43	74.91	66.19	83.91	85.11
300	53.94	53.77	64.15	55.92	71.55	73.49
400	37.07	35.04	37.79	39.13	82.29	73.89
500	26.40	23.18	21.11	31.57	73.19	75.04
600	45.44	44.34	50.87	66.34	84.10	85.28
700	54.76	54.68	65.43	75.61	95.26	95.77
800	31.34	28.67	28.83	72.17	91.11	91.87
900	53.94	53.77	64.15	68.66	86.89	87.91
1000	63.45	64.34	79.01	66.55	84.35	85.51

**Fig. 8:**
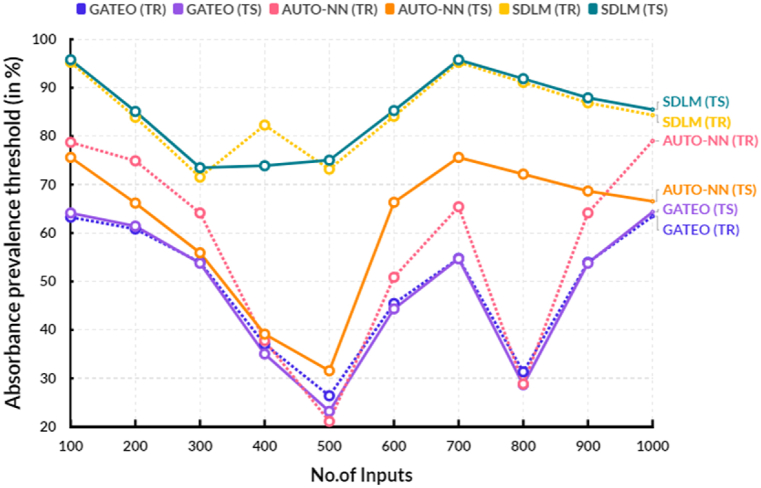
Absorbance prevalence threshold.

Fig. 8 shows the comparison of absorbance prevalence threshold between the existing and proposed models. In a computational range, the proposed solar deep learning model (SDLM) reached 75.04 % of testing and 73.19 % of training results of absorbance prevalence threshold. In the same range, the existing Genetic algorithm based techno-economic optimization (GATEO) reached 23.18 % of testing and 26.40 % of training results and AUTO-Encoder Based Neural-Network (AUTO-NN) reached 31.57 % of testing and 21.11 % of training results of absorbance prevalence threshold. The proposed model’s absorbance prevalence threshold has increased compared with existing models because it takes into account a variety of factors, such as the amount of absorbance per incident photon, the number of incident photons, the light source, the distance of the sample from the detector, and other environmental and sample-specific parameters. This helps to ensure that readings are accurate and reliable, and that absorbance readings can be compared across different optical systems. By accounting for these factors, the proposed threshold increases the accuracy of the readings, allowing for a more reliable comparison between different optical systems.

### 6.5 Computation of critical success index (CSI)

The Critical Success Index (CSI) for thermal energy harvesting is a measure of how successful a certain harvesting process is in terms of its thermal output. It is essentially a measure of the efficiency of the thermal energy harvesting process. It can be calculated by dividing the total thermal energy output by the total energy input. The higher the CSI value, the more efficient the thermal energy harvesting process is. To improve the efficiency of thermal energy harvesting, various techniques can be used such as optimizing the design of the harvesting system, selecting materials with higher thermal conductivity, and improving the thermal insulation.

(15) $$CSI=\frac{P_{t}}{P_{t}+N_{f}+P_{f}}$$

The following Table 6, provides the comparison of critical success index between the existing and proposed models.

**Table 6:** Comparison of critical success index (in %).

No.of Inputs	GATEO (TR)	GATEO (TS)	AUTO-NN (TR)	AUTO-NN (TS)	SDLM (TR)	SDLM (TS)
100	65.29	48.97	88.70	68.35	83.44	90.81
200	47.29	54.79	87.88	67.69	93.81	89.84
300	45.70	53.77	86.39	66.48	91.98	88.07
400	61.72	42.30	82.14	65.53	69.48	92.09
500	59.97	47.30	81.13	64.68	78.09	90.81
600	58.51	46.39	68.11	53.81	76.52	74.35
700	42.37	51.89	67.48	53.29	86.02	73.55
800	40.95	50.93	66.34	52.34	84.35	72.11
900	46.22	69.19	98.16	62.17	97.43	77.06
1000	44.67	67.90	96.49	61.06	95.54	75.54

**Fig. 9:**
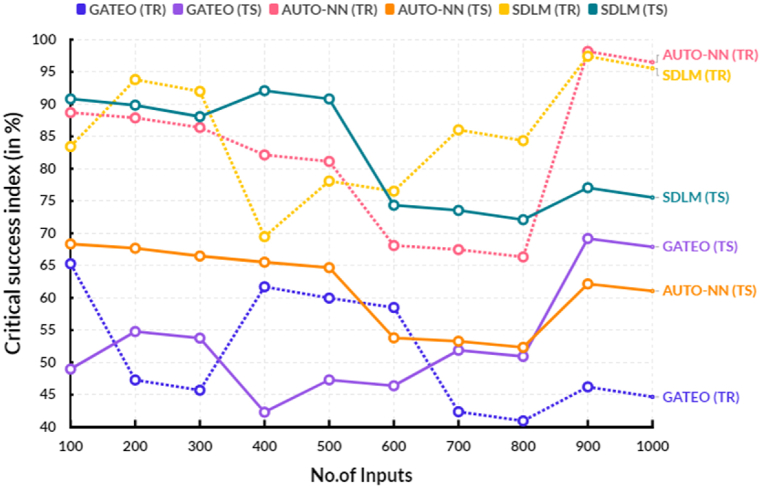
Critical success index.

Fig. 9 shows the comparison of critical success index between the existing and proposed models. In a computational range, the proposed solar deep learning model (SDLM) reached 90.81 % of testing and 78.09 % of training results of critical success index. In the same range, the existing Genetic algorithm based techno-economic optimization (GATEO) reached 47.30 % of testing and 59.97 % of training results and AUTO-Encoder Based Neural-Network (AUTO-NN) reached 64.68 % of testing and 81.13 % of training results of critical success index. The proposed model’s critical success index has increased because it takes into consideration more factors than the existing models. For example, the proposed model accounts for the size of the organization, the impact of action taken, and the strategic alignment of the initiative. This additional information allows the model to more accurately measure the success of an initiative or project. Additionally, the proposed model can be used to evaluate initiatives across an entire organization, rather than just individual departments, divisions, or teams. In doing so, it provides a more holistic view of the organization’s performance. By capturing a broader range of data and taking into account more aspects of the initiative, the proposed model’s data-driven approach allows it to produce a more accurate and comprehensive CSI. Table 7 provides the overall comparison between the existing and proposed models.

**Table 7:** Overall comparison (in %).

Parameters	GATEO (TR)	GATEO (TS)	AUTO-NN (TR)	AUTO-NN (TS)	SDLM (TR)	SDLM (TS)
AR_FP_	41.64	76.96	69.29	66.68	91.72	83.22
DR_FA_	61.72	42.30	82.14	65.53	81.48	69.88
OR_FA_	41.66	48.77	44.91	45.61	72.08	81.40
P_th_	26.40	23.18	21.11	31.57	73.19	75.04
CSI	59.97	47.30	81.13	64.68	78.09	90.81
						

**Fig. 10:**
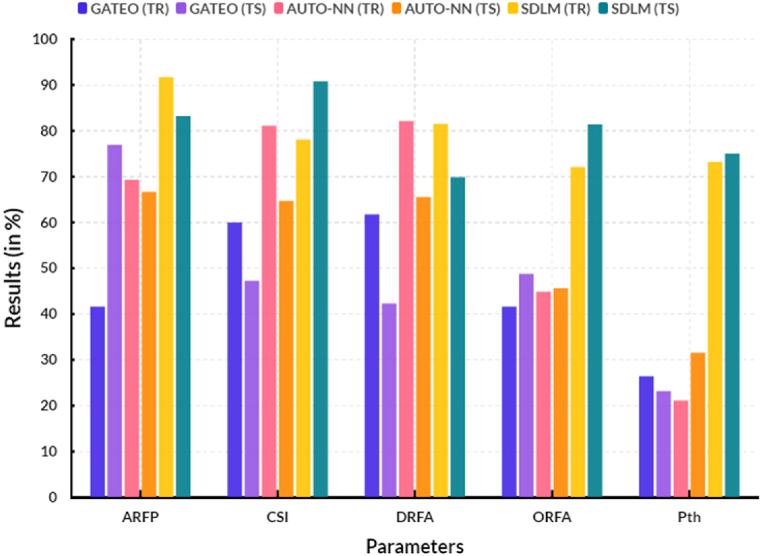
Overall comparison.

Fig. 10 shows the overall comparison results between the existing and proposed models. In a computational range, the proposed solar deep learning model (SDLM) reached 83.22 % of testing and 91.72 % of training results of false positive absorption rate, 69.88 % of testing and 81.48 % of training results of false absorption discovery rate, 81.40 % of testing and 72.08 % of training results of false absorption omission rate, 75.04 % of testing and 73.19 % of training results of absorbance prevalence threshold, and 90.81 % of testing and 78.09 % of training results of critical success index. Table 8 shows the comparative enhancement of proposed model.

**Table 8:** Comparative enhancement.

Parameters	GATEO [2]	AUTO-NN [3]	SDLM [Proposed]
Absorptivity	Low	Medium	High
Emissivity	Medium	High	Low
Energy Consumption	Medium	Medium	Low
Power Utilization	High	Medium	Low
Energy Efficiency	Medium	Medium	High

Once an efficient solar absorber is designed and integrated into a solar energy system, it can be used to generate electricity or to provide hot water for a variety of uses. The thermal energy produced by the absorber can be used for cooling and heating, as well as for driving thermoelectric generators. Additionally, the thermal energy can be stored for later use in thermal batteries or hot water tanks. The use of an efficient solar absorber for thermal energy harvesting is a cost-effective way to generate electricity or provide hot water. The use of an efficient solar absorber can help reduce energy costs and improve the efficiency of a solar energy system. Additionally, the use of an efficient solar absorber can help reduce the environmental impact of energy generation, as it does not generate any emissions or pollutants. The use of an efficient solar absorber for thermal energy harvesting is an important component of a solar energy system. An efficient solar absorber can help reduce energy costs and improve the efficiency of a solar energy system. Additionally, the use of an efficient solar absorber can reduce the environmental impact of energy generation, as it does not generate any emissions or pollutants.

## 7 Conclusion

Solar thermal energy harvesting is an important renewable energy source which has become increasingly popular in recent years. It involves capturing, storing, and using solar energy for various purposes, such as heating, cooling, and electricity production. One of the most important aspects of solar thermal energy harvesting is the maximum power density of the efficient solar absorber. The maximum power density of an efficient solar absorber refers to the maximum amount of energy that can be absorbed per unit area. The efficiency of a solar absorber is determined by its ability to absorb and convert solar energy into heat. There are several factors which affect the efficiency of a solar absorber, such as the material used, the reflectivity of the material, the emissivity of the material, and the surface area of the material. In a computational range, the proposed solar deep learning model (SDLM) reached 83.22 % of testing and 91.72 % of training results of false positive absorption rate, 69.88 % of testing and 81.48 % of training results of false absorption discovery rate, 81.40 % of testing and 72.08 % of training results of false absorption omission rate, 75.04 % of testing and 73.19 % of training results of absorbance prevalence threshold, and 90.81 % of testing and 78.09 % of training results of critical success index. In order to maximize the efficiency of a solar absorber, it is necessary to select the right material and optimize the design of the absorber. Various materials have been used to create efficient solar absorbers, such as aluminum, copper, and steel. However, the most efficient material for a solar absorber is black carbon, which has a high absorption coefficient and a low reflectivity. The surface area of the absorber is also important, as it affects the amount of energy that can be absorbed. In general, larger surface areas will lead to higher efficiencies. In the near future, multi-object deep learning models could be used to optimize the efficiency and performance of the absorbers in accordance with a variety of temperature, surface, weather, and many other conditions. Additionally, AI-driven financial analytic models could be developed to effectively evaluate the economic feasibility of deploying such technology. In the long-term, the development of more intelligent deep learning models could also enable the integration of solar absorbers into various industrial processes which optimize and control energy usage across production processes. Further research could be directed at the development of advanced, automated controls through solar deep learning models in order to ensure the optimal levels of energy harvest.

## CRediT authorship contribution statement

**Ammar Armghan:** Writing – review & editing, Writing – original draft, Software, Investigation, Conceptualization. **Jaganathan Logeshwaran:** Writing – review & editing, Writing – original draft, Software, Investigation, Conceptualization. **S. Raja:** Writing – review & editing, Writing – original draft, Investigation. **Khaled Aliqab:** Writing – review & editing, Writing – original draft, Investigation. **Meshari Alsharari:** Writing – review & editing, Writing – original draft, Investigation. **Shobhit K. Patel:** Writing – review & editing, Writing – original draft, Investigation.

## Declaration of competing interest

The authors declare that they have no known competing financial interests or personal relationships that could have appeared to influence the work reported in this paper.
